# Properties of Biomimetic Artificial Spider Silk Fibers Tuned by PostSpin Bath Incubation

**DOI:** 10.3390/molecules25143248

**Published:** 2020-07-16

**Authors:** Gabriele Greco, Juanita Francis, Tina Arndt, Benjamin Schmuck, Fredrik G. Bäcklund, Andreas Barth, Jan Johansson, Nicola M. Pugno, Anna Rising

**Affiliations:** 1 Laboratory of Bio-Inspired, Bionic, Nano, Meta, Materials & Mechanics, Department of Civil, Environmental and Mechanical Engineering, University of Trento, Via Mesiano 77, 38123 Trento, Italy; gabriele.greco-2@unitn.it; 2 Department of Neurobiology, Care Sciences and Society, Karolinska Institutet, Neo, 14186 Huddinge, Sweden; juanita.francis@ki.se (J.F.); tina.arndt@ki.se (T.A.); benjamin.schmuck@ki.se (B.S.); fredrik.backlund.2@ki.se (F.G.B.); janne.johansson@ki.se (J.J.); 3 Department of Biochemistry and Biophysics, The Arrhenius Laboratories for Natural Sciences, Stockholm University, 10691 Stockholm, Sweden; barth@dbb.su.se; 4 School of Engineering and Materials Science, Queen Mary University of London, Mile End Road, London E1 4NS, UK; 5 Department of Anatomy, Physiology and Biochemistry, Swedish University of Agricultural Sciences, 75007 Uppsala, Sweden

**Keywords:** fiber, tensile testing, mechanical properties, spinning

## Abstract

Efficient production of artificial spider silk fibers with properties that match its natural counterpart has still not been achieved. Recently, a biomimetic process for spinning recombinant spider silk proteins (spidroins) was presented, in which important molecular mechanisms involved in native spider silk spinning were recapitulated. However, drawbacks of these fibers included inferior mechanical properties and problems with low resistance to aqueous environments. In this work, we show that ≥5 h incubation of the fibers, in a collection bath of 500 mM NaAc and 200 mM NaCl, at pH 5 results in fibers that do not dissolve in water or phosphate buffered saline, which implies that the fibers can be used for applications that involve wet/humid conditions. Furthermore, incubation in the collection bath improved the strain at break and was associated with increased β-sheet content, but did not affect the fiber morphology. In summary, we present a simple way to improve artificial spider silk fiber strain at break and resistance to aqueous solvents.

## 1. Introduction

In recent decades, spider silks and their extraordinary mechanical and biological properties have gained interest from the scientific community as well as from the industry [1,2,3,4]. Spider silk is envisioned as an interesting material for applications in tissue engineering [2,5,6,7,8,9,10,11], the textile industry [12,13] and as reinforcement in composites [14]. Unfortunately, spiders produce small amounts of silk and they are difficult to farm due to their cannibalistic, predatory, and solitary nature [15]. To overcome this problem, production of recombinant silk proteins (spidroins) in heterologous hosts has been seen as a plausible solution that also would be beneficial from an environmental point of view [16,17]. Several protocols for recombinant spidroin production in different prokaryotic and eukaryotic systems have been published [18,19,20,21,22,23,24] and there are even protocols for spidroin production in mammalian hosts [25,26,27]. Common problems encountered when producing spidroins in these hosts have been low protein yields and premature aggregation during expression, which resulted in aggregated target proteins that required solubilization in harsh solvents such as hexafluoroisopropanol (HFIP) [23,28]. Such conditions denatured the proteins and were incompatible with a biomimetic spinning procedure [16].

**Figure 1:**
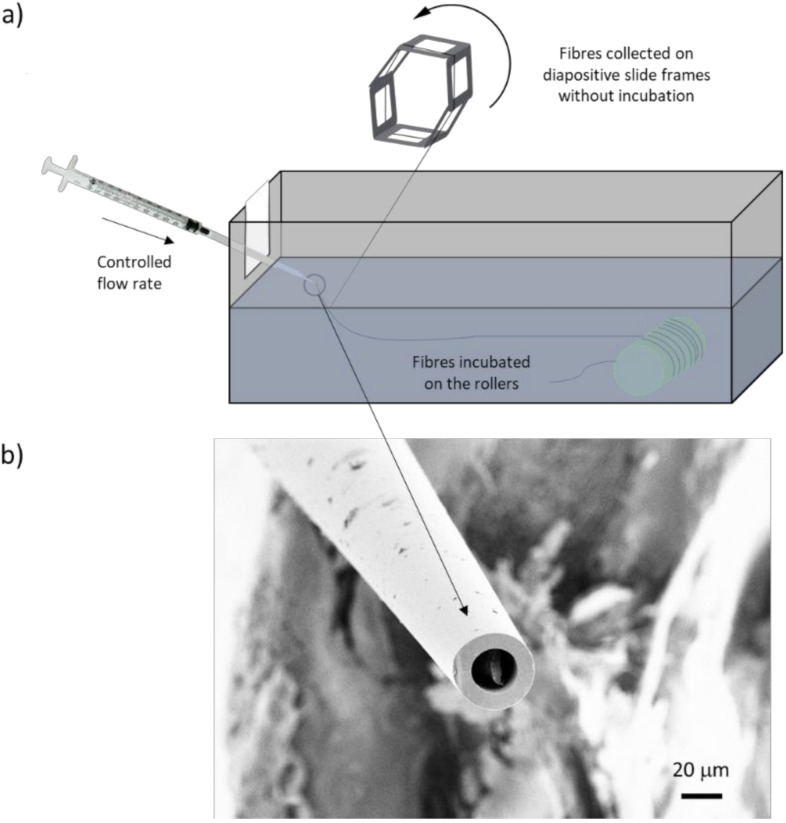
(**a**) Schematic diagram of the spinning setup. (**b**) SEM picture of a capillary used to spin the artificial spider silk fibers.

Spiders are able to keep the spidroins soluble during storage in the glands at an impressive concentration of 30–50% (w/v) [29], and they convert the protein solution into solid fibers using a drop in pH to <5.7 and shear forces [30]. Recently, this high solubility has been achieved for designed recombinant spidroins that were purified from *E. coli* and kept, in an aqueous buffer, at pH 8.0 [31,32]. Moreover, these recombinant spidroins have been spun into continuous fibers in biomimetic spinning setups where the fibers were formed by extrusion of a highly concentrated protein solution into a collection bath (pH 5.0 aqueous buffer) [31,32,33] (Figure 1). Unfortunately, the artificially spun fibers produced in this way do not match the native fibers in terms of mechanical properties and they are sensitive to wet environments [31,32,33].

In this work, we show that the strain at break of biomimetic artificial spider silk fibers can be improved by means of increased incubation time in the collection bath. Notably, after ≥5 h incubation, the fibers do not dissolve in deionized water (dH_2_O) or phosphate buffered saline (PBS). On the basis of our findings we present a simple protocol to tune the mechanical properties and avoid water-solubility of biomimetic artificial spider silk fiber.

## 2. Results

The miniature spidroin NT2RepCT was produced and concentrated to 300 mg/mL and spun into fibers essentially as previously described [31]. Briefly, a syringe pump was used to extrude the protein solution through a pulled glass capillary into an aqueous buffer at pH 5.0 (500 mM NaAc and 200 mM NaCl, Figure 1a). The fibers were either immediately taken up from the collection bath onto a motorized wheel with diapositive slide frames attached (0 h incubation) or collected onto a roller that was submerged in the collection bath (Figure 1a). The immersed roller was left in the bath for fixed periods of time ranging between 1 and 48 h, where after it was removed from the bath, the fibers were collected from the roller, dried in air, and tested for water solubility, as well as characterized by tensile testing using Fourier transform infrared (FTIR) spectroscopy, light microscopy, and scanning electron microscopy (SEM). 

First, we investigated the resistance of fibers to aqueous solvents. Dried fibers were placed in either dH_2_O or PBS and their integrity was observed immediately by the naked eye after 24 h, 48 h, and 1 week. Fibers that were collected immediately and the fibers that were incubated for 1 and 2 h, respectively, shrunk and dissolved immediately when they came into contact with dH_2_O or PBS. In contrast, fibers that were incubated in the collection bath for 12 and 48 h decreased slightly in length immediately upon contact with the aqueous solvents but remained intact for more than 48 h and 1 week in dH_2_O and PBS, respectively. To further pinpoint the timepoint for conversion to a more resistant type, we incubated a second set of fibers for 3, 5, 5.5, and 6 h in the collection bath before performing the solubility assays. The results showed that ≤3 h incubation resulted in fibers that dissolved in aqueous solvents but incubation in the collection bath for ≥5 h resulted in fibers that remained intact (Table S1). 

**Figure 2:**
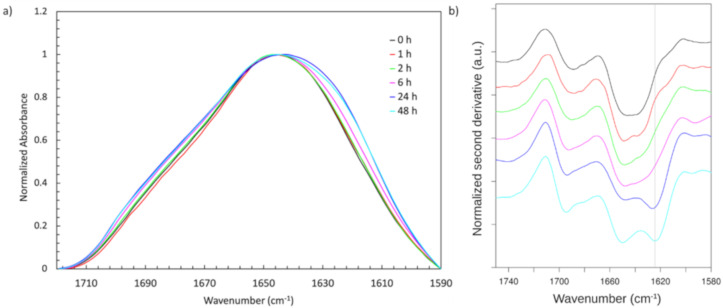
(**a**) Averaged, normalized, and baseline-subtracted absorbance spectra in the amide I region of NT2RepCT fibers incubated in spinning buffer for 0, 1, 2, 6, 24, and 48 h; (**b**) Normalized second derivatives of the absorbance spectra of NT2RepCT fibers incubated in spinning buffer for 0 h (black), 1 h (red), 2 h (green), 6 h (purple), 24 h (blue), and 48 h (turquoise). The gray vertical line indicates the position of the β-sheet band observed after 24 and 48 h of incubation.

FTIR spectroscopy of the NT2RepCT fibers showed an increased absorbance around 1630–1620 cm^−1^ in the amide I region for fibers incubated for 6 h or longer, which is consistent with increased β-sheet content (Figure 2a). To explore this further, second derivative spectra were generated in which negative bands indicated the positions of component bands in the respective absorbance spectrum (Figure 2b). Incubation in the spinning buffer led to clear changes in the second derivative spectra, i.e., a new component appeared near 1625 cm^−1^ and the high wavenumber band near 1695 cm^−1^ became more distinct. These features are typical for antiparallel β-sheets, which are rather flat and extended [34,35,36]. Double difference spectra (absorbance at time × minus absorbance at time 0, not shown) showed a decrease in absorbance near 1650 cm^−1^ where the main components of the spectrum at 0 h incubation time were observed in the second derivative spectrum. This indicated that the initial structure was transformed to an extended β-sheet structure upon prolonged incubation.

**Figure 3:**
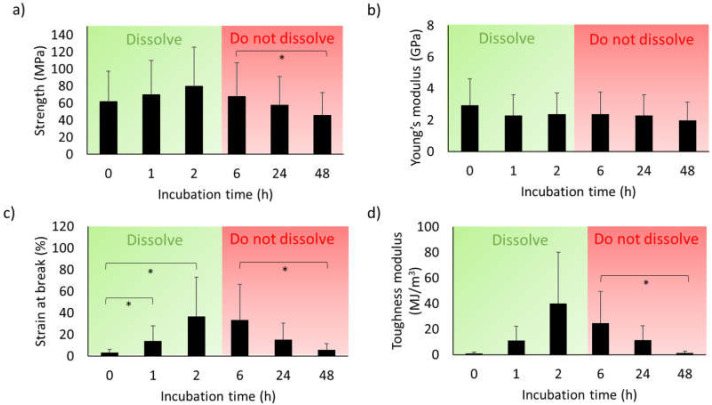
Mechanical properties of the fibers vs. the incubation time in the collection bath. (**a**) Strength (MPa); (**b**) Young’s modulus (GPa); (**c**) Strain at break (mm/mm); (**d**) toughness modulus (MJ m^−3^). Green indicates that the fibers dissolved and red that they stayed intact when submerged in either dH_2_0 or PBS. Stars indicate that the differences were statisitically significant (*p* < 0.05). Depending on the typology, the following numbers of fibers were tested: 17 for 0 h, 6 for 1 h, 5 for 2 h, 34 for 6 h, 17 for 24 h, and 10 for 48 h. * indicates *p* < 0.05.

Next, we analyzed the mechanical properties of the fiber. The incubation time seemed to not affect the strength or Young’s modulus significantly over the course of the first 24 h of incubation (Figure 3a,b and Table S1). In pairwise comparisons among the groups, we noticed a significant difference only between the fibers incubated for 6 and 48 h. Notably, the strain at break increased significantly during the first 2 h of incubation (Figure 3c and Table S1). The mean strain values obtained for fibers incubated for 0, 1, and 2 h more than doubled for each hour of incubation, thereafter the strain remained unchanged for the 6 and 24 h samples. The fibers that were incubated for 6 and 24 h did not show any significantly altered properties as compared with 2 h incubation for any of the investigated mechanical parameters (Figure 3a–d and Table S1). In contrast, the fibers that were incubated for 48 h presented a lower strain at break and toughness modulus as compared with the fibers incubated for 6 h. The qualitative analysis of the stress–strain graphs showed that, after ≥1 h incubation, a clear yield point emerged, which meant that the fibers become plastic and more deformable (Figure S1). In general, the best performing fibers (obtained after 2–6 h of incubation) had strain values comparable to native silk fibers but ultimate tensile strength was 10–20% of that of most spider silks [37].

**Figure 4:**
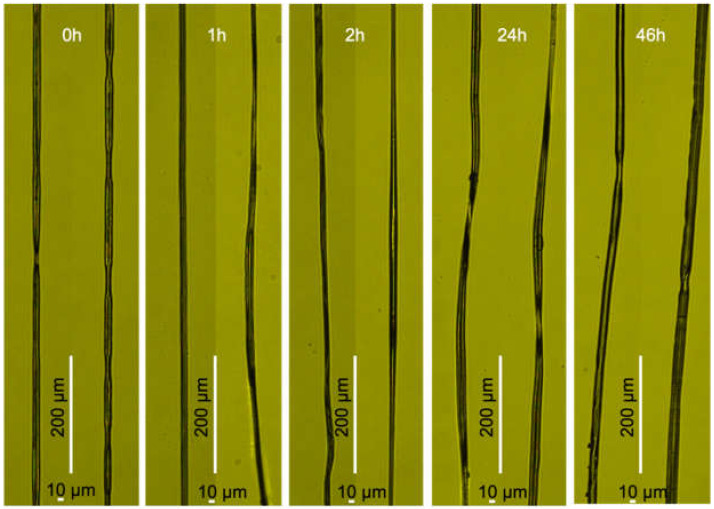
Light microscopy images of fibers with indicated incubation times.

**Figure 5:**
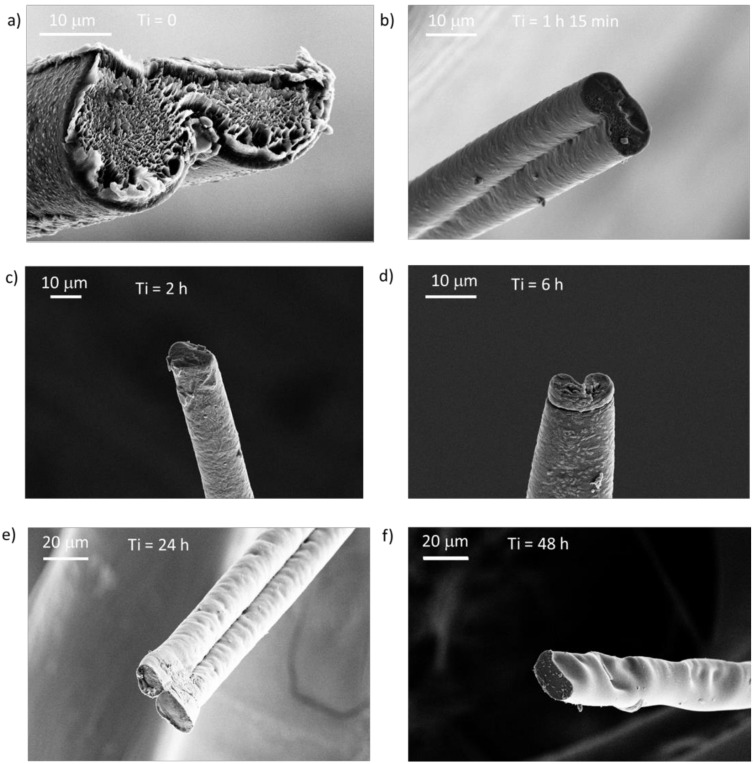
Fracture sections of fibers that were incubated for (**a**) 0 h; (**b**) 1 h and 15 min; (**c**) 2 h; (**d**) 6 h; (**e**) 24 h; (**f**) 48 h in the collection bath.

The fiber morphology was unaffected by both incubation time and method of collection (frames vs. roller). However, we found that most fibers exhibited a pronounced “dual fiber” appearance with a concomitant dumbbell-shaped cross-section (Figure 4, Figure 5, and Figure S3). Some fibers were a bit flattened and had a more elliptical cross-section (Figure 5). The reason for this is not clear, but it is not directly due to a defect in the shape of the capillary nozzle, which was found to be circular (Figure 1b). In addition, the morphology of the fibers was not always uniform along the same fiber.

## 3. Discussion

Herein, we explore possible means to modulate the properties of artificial spider silk fibers by tuning the incubation period in the collection bath. In order to allow incubation of the fibers in the collection bath with minimal fiber handling, we constructed a roller that was motorized and submerged into the collection bath, and optionally the fibers could be immediately collected onto rotating frames in air (Figure 1a). In this manner, fibers were collected at an even and fixed speed.

Incubation times ≥5 h resulted in fibers that were resistant to aqueous environments and these fibers behaved similar to native silk in that they initially contracted upon contact with dH_2_O or PBS but after that remained intact [38]. In contrast, the fibers that were incubated for 3 h or less, almost immediately dissolved in contact with dH_2_O or PBS. Because β-sheet crystalline structures contribute to the stability of silk biomaterials in a wide range of environments, including in water [11], we investigated, using FTIR spectroscopy, if increased β-sheet content could be related to the decrease in fiber solubility. For fibers incubated for 6 h and longer, there was indeed an increased β-sheet content, most likely due to the formation of antiparallel β-sheets, as shown by increased absorbance around 1630–1620 cm^−1^ as compared with fibers incubated for 2 h or less (Figure 2). Thus, the observed increase in β-sheet content coincides with the conversion from soluble to insoluble fibers. It is not known which part of the NT2RepCT protein is responsible for the structural conversion, but previous reports have shown that the C-terminal domain converted to β-sheet structures in response to low pH [30,39] and that the poly-Ala blocks of the repetitive region could form helical or β-strand conformations in the NT2RepCT fiber [34]. The NT is unlikely to contribute to the increased β-sheet content since it is α-helical also at a low pH [40].

The mechanical parameter that was most affected by incubation in the collection bath was strain at break (Figure 3). Fibers that were incubated for 1–2 h showed significantly increased strain as compared with the fibers that were immediately collected, but increased incubation time beyond 2 h did not improve the properties further, and after 48 h, we detected a significant decrease in strain as compared with the fibers incubated for 6 h (Figure 3c). Therefore, the best mechanical properties were observed as early as after 2 h of incubation, but water resistance requires incubation for at least 5 h. The increase in strain at break observed after 1 and 2 h of incubation, respectively, did not correlate with any detectable changes in overall secondary structure content as observed by FTIR spectroscopy (Figure 2 and Figure 3). The increase in β-sheet content observed after 24 and 48 h (Figure 2) is linked to decreased strain, which fits with the current model that random coil, turn, and helical structures contribute to fiber extensibility [41,42,43]. However, there was no observed increase in tensile strength of the fiber which would have been expected to result from increased β-sheet content [44].

The changes in mechanical properties and water resistance were not accompanied by any significant morphological change as observed by light microscopy or SEM (Figure 4 and Figure 5). However, an important observation was that the fibers had a “dual fiber” or flattened appearance (Figure S3), which had implications for the stress calculations (Figure S2). The tensile strength values, shown in Figure 3 and Table S1, were calculated assuming a circular cross-section of the fiber. This meant that if the fibers actually were not circular (but elliptical or dumbbell-shaped) we overestimated the fiber cross-sectional area by a factor of two, and thereby correspondingly underestimated the fiber tensile stress (Figure S2). Artificial silk fibers with dumbbell-shaped cross-sections have also been observed in NT2RepCT spinning setups which, instead of the pulled glass capillary, employed fluorinated ethylenepropylene tubes with round cross-sections [32], as well as other spinning setups and recombinant spidroins [45]. Notably, in these studies the cross-sectional area was also assumed to be circular when calculating tensile strength. By assuming a circular cross-sectional area it avoids overestimation of the mechanical properties, but complicates attempts to understand the basis for the mechanical properties of artificial silk fibers if it does not truly reflect the fiber morphology. The reasons for the observed dual fiber appearance in our spinning setup were not identified, but the the fiber morpholoiges were not a direct result of the shape of the hand-drawn glass capillary, as the nozzle was found to be circular (Figure 1). 

Compared to silk spinning by a spider that happens in fractions of a second, our method requires significantly longer time for the fibers to become resistant to wet environments. This could depend on several factors including differences in flow dynamics, protein size and dope viscosity, ion and pH gradients, etc. In our spinning device, the spidroins were not sheared in the same way as natural spidroins are, since the silk fiber is pulled out from the duct of the spider, not extruded [46]. It is obvious that the spinning method has a significant impact on the properties of the resulting fibers, and increased incubation time combined with post-spin draw could be a promising route forward to achieve both higher strain at break and ultimate tensile strength of artificial fibers [45,47]. In summary, although the molecular mechanisms that are responsible for the observed change in properties remain to be determined, we herein present a feasible and simple way of improving strain at break and the resistance to aqueous solutions of biomimetic silk fibers.

## 4. Conclusions

Making spider silk proteins in heterologous hosts is the most promising route to successful production of artificial spider silk [9]. To obtain truly silk-like fibers, protein production and purification under non-denaturing conditions and subsequent biomimetic spinning methods are likely required [16]. In this work, we present a simple protocol using benign conditions to improve the mechanical properties and water resistance of biomimetically spun artificial spider silk fibers, both of which are important features for biomaterials intended for biomedical applications. 

## 5. Materials and Methods

### 5.1. Preparation of the Spinning Dope

The NT2RepCT protein was produced essentially as described in [31]. Briefly, an overnight culture was prepared using Luria broth (LB) media with 1:1000 kanamycin (70 mg/L) inoculated with a glycerol stock of *Escherichia coli* BL21DE3 harboring the plasmid pT7-6xHis-NT2RepCT. The culture was grown overnight at 30 °C with shaking (200 rpm). One liter baffled shake flasks containing 1 L of LB media with kanamycin (1:1000) were inoculated with 1/100 of overnight culture and cultured at 30 °C with shaking (110 rpm.), until OD_600_ reached 0.6. Subsequently, the temperature was reduced to 20 °C and growth continued until OD_600_ reached 0.8–0.9, and then protein expression was induced by 1 M isopropylthiogalactoside to a final concentration of 0.3 mM. Culturing continued overnight and cells were harvested by centrifugation, 7278× *g* for 20 min. Cells were resuspended in 20 mM Tris pH 8 (40 mL/L culture) and subjected to freezing at −20 °C. Cells were lysed using a cell distributer (T-series Machine, Constant Systems Limited, Daventry, Northants, UK) at 30 kPsi equipped with a recirculating chiller (Neslab ThermoFlex™ 1400, Thermo Scientific, Waltham, MA, USA) set to 4 °C. The lysate was centrifuged at 27,000× *g* at 4 °C for 30 min. Purification was performed using the Ni-NTA column protocol as described in [31], however after loading the supernatants, two wash steps were conducted. The first wash was with 20 mM Tris pH 8 and the second wash was with 5 mM imidazole. The protein was concentrated using an ultrafiltration spin column (Vivaspin 20, GE Healthcare, Chicago, IL, USA) with a 10 kDa molecular weight cutoff, to a high concentration of approximately 300 mg/mL. Spin columns were subjected to 4000× *g* for 20 min repeatedly until the final concentration was reached. To determine the protein concentration, 1 µL of protein was diluted 1000 times, this was performed in triplicate. The absorbance of each sample was measured at 280 nm using a spectrophotometer, the protein concentrations were calculated using the molar extinction coefficient of NT2RepCT, 18,910 M^−1^⋅cm^−1^, and the average mean was recorded. Three batches of concentrated NT2RepCT protein dope were produced and loaded into separate 1 mL syringes with Luer Lok tip (BD, Franklin Lakes, NJ, USA) and frozen at −20 °C.

### 5.2. Artificial Spider Silk Spinning and Incubation

Fiber spinning was performed following a modified protocol designed by Andersson et al. [31]. The syringe containing the protein dope was thawed at room temperature and connected to a 27 G blunt end steel needle (B. Braun, Melsungen, Germany) with an outer diameter of 0.40 mm. Round glass capillaries (G1 Narishige, Tokyo, Japan) with an outer diameter (O.D.) of 1.0 mm and inner diameter (I.D.) of 0.6 mm were pulled using a Micro Electrode Puller (Stoelting Co. 51217, Wood Dale, IL, USA) and cut to a tip diameter of 25–35 µm (Figure 1b). The needle was connected to the capillary by a series of two tubings. The first being a polyethylene tubing (BD Intramedic, Franklin Lakes, NJ, USA) with an O.D. of 1.09 mm and an I.D. of 0.38 mm encasing the needle with a ~5 mm overhang and inserted ~1 cm into the larger polyethylene tubing with an O.D. of 1.65 mm and an I.D. of 0.76 mm of which the capillary was inserted into, ~5 mm. The extensibility of the tubing allowed the components to be fitted together. A neMESYS low pressure (290 N) syringe pump (Cetoni, Korbußen, Germany) was used to extrude the protein into the collection bath (500 mM NaAc and 200 mM NaCl, pH 5 at a flow rate of 17 µL/min). For collection of the fibers, we used a custom made movable bidirectional motorized pulley system in which a circular roller with a 30 mm diameter was attached and submerged into the spinning buffer (Figure 1a). The roller was placed approximately 30 cm from the tip of the capillary and rotated counterclockwise. Extruding fibers were guided along the collection bath using a 1 µL inoculation tube and picked up by the roller. After spinning, fibers were either removed immediately from the roller or were kept on the submerged roller in the spinning buffer for 1, 2, 6, 24, and 48 h, respectively. Fibers were removed from the roller using surgical tweezers at each incubation time and placed on a black plastic sheet to dry. To pinpoint the exact incubation time that gave water resistant fibers, we performed a second series of experiments in which we incubated the fibers for 3, 5, and 5.5 h. To determe the mechanical properties of the 0 h time point, the fibers were immediately collected onto six rotating diapositive slide frames mounted on a motorized wheel. 

### 5.3. Solubility Assay

For each incubation time, two petri dishes were prepared in which one was filled with dH_2_O and the other was filled with filtered 1 × PBS, pH 7.4. The petri dishes were placed on top of a black plastic sheet in order to visualize the fibers. Pieces of dried fibers from the respective incubation times were placed in dH_2_O and 1 × PBS, respectively, and their integrity was observed by the naked eye after 24 h, 48 h, and 1 week.

### 5.4. Fourier Transform Infrared (FTIR) Spectroscopy

Incubated NT2RepCT fibers at the various time points were measured using a Vertex 70 instrument equipped with a Platinum-ATR and an MCT-detector (Bruker, Ettlingen, Germany) to obtain FTIR spectra on single fibers. To provide a stable environment for measuring, the instrument was purged with dry air which minimized water vapor and carbon dioxide interference with the sample spectra. For each sample condition, 6 fibers were measured; 3 fibers were measured in parallel to the infrared beam and the remaining 3 fibers were perpendicular to the beam. Prior to measuring each fibre, a background spectrum (200 scans) was obtained. Two hundred scans were recorded for each fiber at a resolution of 2 cm^−1^. The spectra were analyzed using Spectragryph, in which the 6 spectra were averaged, baseline subtracted, and then normalized to a maximum absorbance of 1 in the 1720–1590 cm^−1^ region. The second derivative spectra were processed with OPUS 5.5.

### 5.5. Tensile Tests

The fibers were mounted on a 1 cm square window cut in a paper frame following the procedure of Greco et al. [48]. The fibers were glued onto the frames using double sided tape. For tensile tests, a 5943-Instron tensile tester machine with a 5 N load cell was used. The imposed displacement speed was 60 mm min^−1^. The engineering stress was calculated by dividing the measured force by the section of each tested thread. We measured the maximum diameter along the fiber with an optical microscope [49], and we measured it 5 times for each fiber by using the mean value for the calculation of the section’s area, which was assumed to be circular. The engineering stress was obtained by dividing the force by the cross-sectional area. The engineering strain was obtained by dividing the total displacement by the gauge length. The Young’s modulus was found by measuring the slope of the stress–strain curve in the initial linear elastic part. The toughness modulus was obtained by calculating the area under the stress–strain curve. We did not make any selection of the data obtained, the values for all fibers tested are shown in Table S1.

### 5.6. Statistical Analysis

#### 5.6.1. ANOVA Analysis

One-way analysis of variance was performed to compare the mechanical properties of the fibers incubated in the spinning buffer for different times (pairwise comparison among groups). The two-tailed *p*-values were computed in Matlab^®^ (MathWorks, Natick, MA, USA) and the significance was considered when *p* < 0.05. For more details see Supplementary Materials.

#### 5.6.2. Light Microscopy

Light microscopy images were collected using a Nikon eclipse TE300 inverted microscope equipped with a DFK 33UX249 camera from Imagingsource. For the 0 h incubation sample, the frames used to collect the fibers were placed on a glass microscopy slide and images recorded following 2 h of drying in air. For the 1, 2, 24, and 46 h samples fibers were removed from the submerged roller and allowed to dry in air for 2 h prior to being secured to glass microscopy slides using nail polish and subsequent recording of images. 

#### 5.6.3. Scanning Electron Microscopy

We investigated the morphology of the fibers with FE-SEM using a Zeiss Supra 40. The metallization was made by a sputtering machine Quorum Q150T with sputtering mode Pt/Pd 80:20 for 5 min. 

## Supplementary Materials

The Supplementary Materials are available online.
