# Supplementary material for: Properties of Biomimetic Artificial Spider Silk Fibers Tuned by PostSpin Bath Incubation
Source: Molecules. 2020 Jul 16;25(14):3248. doi: 10.3390/molecules25143248 (PMC7397010; doi:10.3390/molecules25143248)
Supplement: Supplementary file 1 [file molecules-25-03248-s001.pdf]

molecules

MDPI

Supplementary information

Properties of Biomimetic Artificial Spider Silk Fibers Tuned by PostSpin Bath Incubation

Gabriele Greco [1,†], Juanita Francis [2,†], Tina Arndt [2], Benjamin Schmuck [2], Fredrik G. Bäcklund [2], Andreas Barth [3], Jan Johansson [2], Nicola M. Pugno [1,4,*] and Anna Rising [2,5,*]

[1] Laboratory of Bio-Inspired, Bionic, Nano, Meta, Materials & Mechanics, Department of Civil, Environmental and Mechanical Engineering, University of Trento, Via Mesiano 77, 38123 Trento, Italy; gabriele.greco-2@unitn.it

[2] Department of Neurobiology, Care Sciences and Society, Karolinska Institutet, Neo, 14186 Huddinge, Sweden; juanita.francis@ki.se (J.F.); tina.arndt@ki.se (T.A.); benjamin.schmuck@ki.se (B.S.); fredrik.backlund.2@ki.se (F.G.B.); janne.johansson@ki.se (J.J.)

[3] Department of Biochemistry and Biophysics, The Arrhenius Laboratories for Natural Sciences, Stockholm University, 10691 Stockholm, Sweden; barth@dbb.su.se

[4] School of Engineering and Materials Science, Queen Mary University of London, Mile End Road, London E1 4NS, UK

[5] Department of Anatomy, Physiology and Biochemistry, Swedish University of Agricultural Sciences, 75007 Uppsala, Sweden

† These authors contributed equally.

* Correspondence: nicola.pugno@unitn.it (N.M.P.); anna.rising@ki.se (A.R.); Tel./Fax: +39 0461 282525 /+39 0461 282599 (N.M.P.); Tel./Fax: +46 704 10 6997 (A.R.)

Academic Editor: Yasumoto Nakazawa
Received: 27 June 2020; Accepted: 13 July 2020; Published: 16 July 2020

ANOVA Analysis

One-way Analysis of Variance was performed to compare the mechanical properties of the fibers of two groups incubated in the spinning buffer for different times. The parameters used to verify the null hypothesis, i.e. all the data sets come from the same distribution and have the same mean value, were:

$$SSQ_a = \sum_{g=1}^{G} n_g \left(m_g - m\right)^2$$

$$SSQ_e = \sum_{g=1}^{G} \sum_{j=1}^{n_g} \left(x_{gj} - m_g\right)^2$$

Where G is the number of different samples under consideration, n_g is the number of tests of the same sample, m is the mean value of all the data, m_g is the mean value within the group (i.e., sample)x is the single quantity value. These sums of squares were used to compute the T value

$$T = \frac{\frac{SSQ_a}{G-1}}{\frac{SSQ_e}{n-G}}$$

that has been compared with the ideal value of the Fisher function F with a significance level of 5%. If $T > F$, we reject the null hypothesis and thus we can consider the difference among the data set as significant (i.e., the difference is due to intrinsic differences among the samples and not a consequence of internal variance). This parameter and the two-tailed p-value were computed with the support of Matlab®.

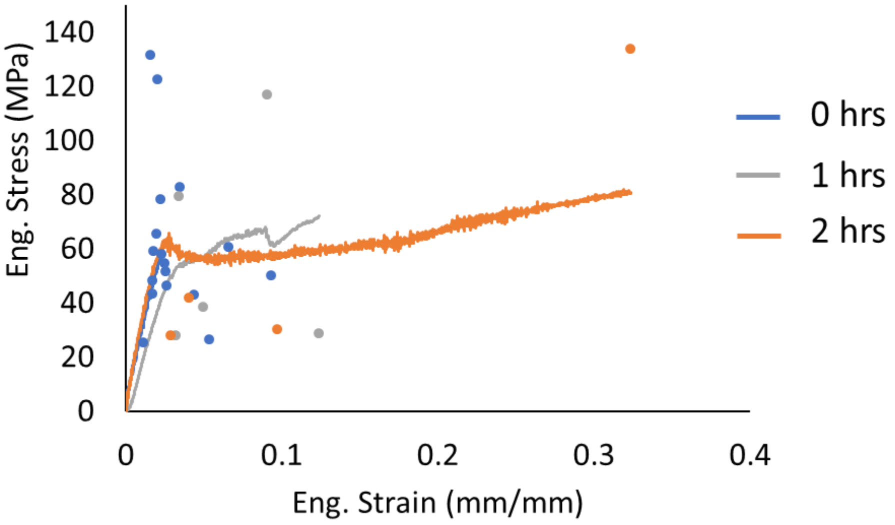

Figure S1. Stress and strain values at failure for all fibers incubated for 0 (blue),1 (grey) and 2h (orange) displayed as dots. Representative stress-strain curves for fibers incubated for 1 or 2 h are also shown. Incubation for ≥1h lead to fibers' stress strain curves displaying a yielding point.

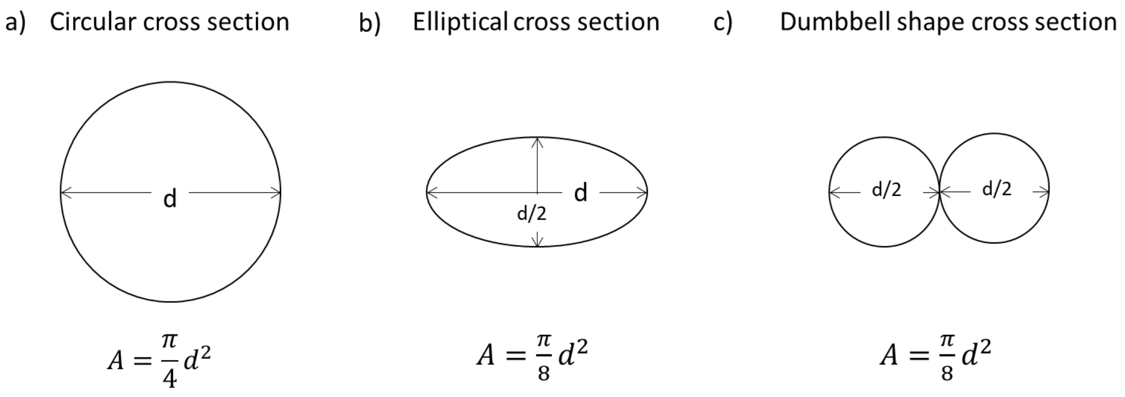

Figure S2. Same diameter but different cross-sectional shape results in a factor 2 difference in calculated cross-sectional area of the fibres. a) Circular shape, b) elliptical and c) dumbbell shape.

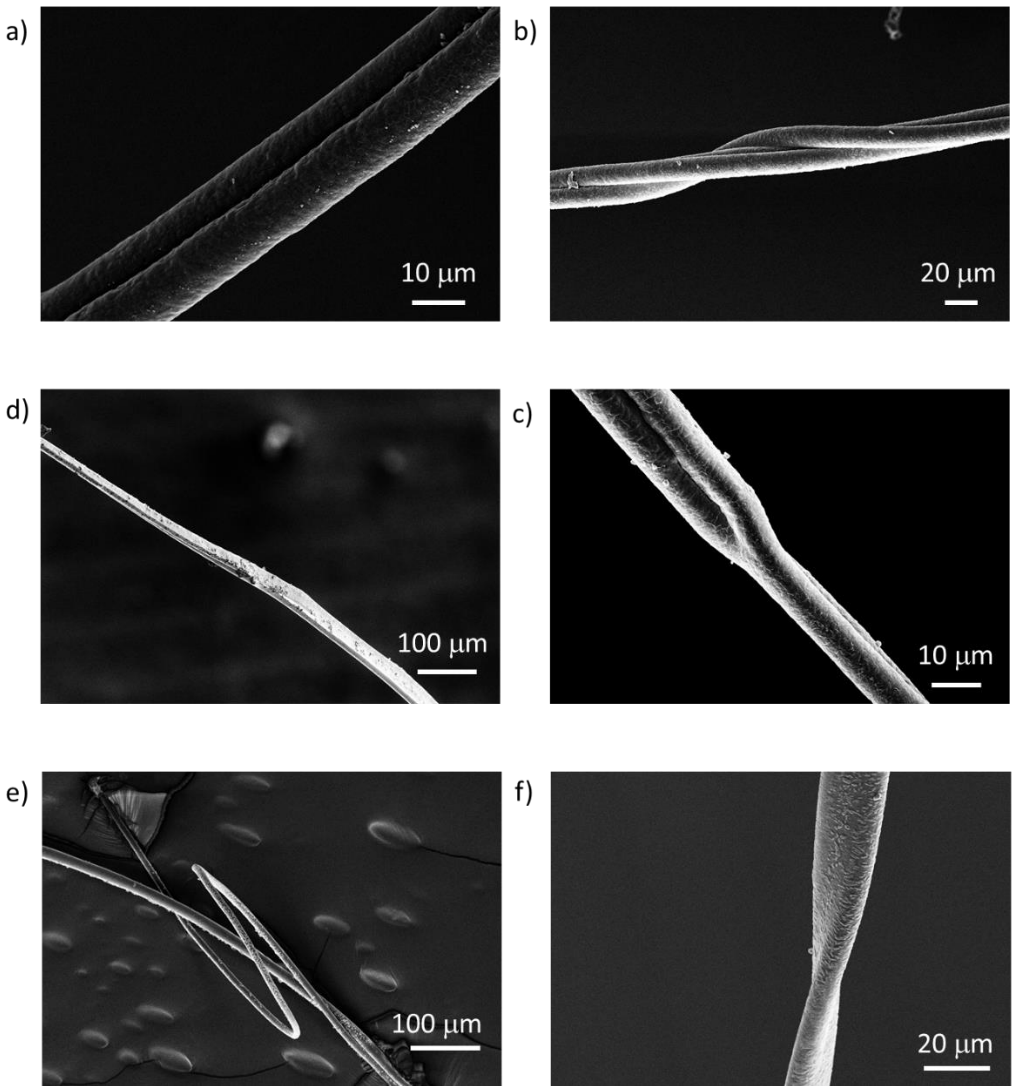

Figure 3. SEM images of fibers with a dumbbell or elliptical cross section shaped cross section.

Table S1. Mechanical properties calculated assuming a circular cross-sectional area and integrity of fibers in aqueous solvents.

Inc. time (h)	Nr. samples	Diameter (m)	Strain at break (mm/mm)	Strength (MPa)	Young's modulus (GPa)	Toughness modulus (MJ/m^3)	Solubility in dH2O	Solubility in PBS
0	17	15 ± 2.4	0.03 ± 0.02	62 ± 29	2.9 ± 2.0	1.0 ± 0.7	YES	YES
1	6	23 ± 10	0.14 ± 0.19	70 ± 44	2.3 ± 1.0	11 ± 20	YES	YES
2	5	15 ± 7	0.37 ± 0.56	80 ± 64	2.4 ± 1.7	40 ± 70	YES	YES
3	8	29 ± 12	0.07 ± 0.05	38 ± 17	1.2 ± 0.5	1.1 ± 0.7	YES	YES
5	29	22 ± 6	0.05 ± 0.04	54 ± 36	1.9 ± 1.0	1.8 ± 2.5	NO	NO
5.5	19	22 ± 11	0.11 ± 0.13	48 ± 28	1.8 ± 1.1	4.9 ± 8.0	NO	NO
6	34	16 ± 8	0.33 ± 0.39	68 ± 30	2.4 ± 1.1	25 ± 34	NO	NO
24	17	15 ± 5	0.15 ± 0.23	58 ± 31	2.3 ± 1.1	11 ± 22	NO	NO
48	10	23 ± 7	0.05 ± 0.03	46 ± 28	2.0 ± 1.3	1.4 ± 1.5	NO	NO
